# Structured Smoking Cessation Training for Medical Students: A Prospective Study

**DOI:** 10.1093/ntr/ntw191

**Published:** 2016-08-24

**Authors:** Ronja Herold, Sarah Schiekirka, Jamie Brown, Alex Bobak, Andy McEwen, Tobias Raupach

**Affiliations:** ^1^Department of Cardiology and Pneumology, University Medical Centre Göttingen, Göttingen, Germany;; ^2^Division of Medical Education Research and Curriculum Development, University Medical Centre Göttingen, Göttingen, Germany;; ^3^Cancer Research UK Health Behaviour Research Centre, University College London, London, UK;; ^4^Wandsworth Medical Centre, London, UK;; ^5^National Centre for Smoking Cessation and Training, London, UK

## Abstract

**Introduction:**

Physician adherence to guideline recommendations regarding the provision of counseling and support for smokers willing to quit is low. A lack of training during undergraduate medical education has been identified as a potential cause. This prospective intervention study evaluated a novel teaching module for medical students.

**Methods:**

As part of a 6-week cardiovascular course, 125 fourth-year undergraduate medical students received a multimodal and interactive teaching module on smoking cessation, including online learning material, lectures, seminars, and practical skills training. Short- and medium-term effects on knowledge, skills, attitudes, and self-reported practice were measured using written examinations and an objective structured clinical examination at the end of the module and 6 months later. Results were compared to data obtained from a historical control cohort (*n* = 70) unexposed to the intervention.

**Results:**

At the 6-month follow-up, scores in the knowledge test were significantly higher in the intervention than the control group (61.1% vs. 51.7%; *p* < .001). A similar pattern was observed in the objective structured clinical examination (71.5% vs. 60.5%; *p* < .001). More students in the intervention than control group agreed that smoking was a chronic disease (83.1% vs. 68.1%; *p* = .045). The control group was more likely to report recording smoking status (*p* = .018), but no group difference was detected regarding the report of advising to quit (*p* = .154).

**Conclusions:**

A novel teaching module for undergraduate medical students produced a sustained learning outcome in terms of knowledge, skills, and attitudes but not self-reported practice.

**Implications:**

Studies across the world have identified considerable knowledge gaps and deficits in practical training with regard to smoking cessation counseling in undergraduate medical students. This paper describes a teaching intervention informed by current recommendations for the design of educational activities aimed at enabling medical students to deliver adequate behavior change counseling. The teaching module was tailored to the needs of a specific healthcare system. Given its effectiveness as demonstrated in this prospective study, a rollout of this intervention in medical schools might have the potential to substantially improve medical students’ knowledge, skills, and attitudes in relation to smoking cessation counseling.

## Introduction

Smoking accounts for 20%–30% of total cardiovascular mortality,^1^ and smoking cessation is among the most effective interventions following a myocardial infarction.^2,3^ A recent study involving biochemical validation of smoking status showed that continuous abstinence is associated with an up to 80% reduction in mortality in this patient group.^4^ Despite hospitalization for cardiovascular disease being a teachable moment for some patients,^5^ successful smoking cessation requires intensive support: According to a Cochrane analysis, interventions for patients hospitalized for cardiovascular disease need to start during inpatient treatment and must be continued for at least 1 month in order to be effective.^6^ Thus, initial counseling during hospitalization is crucial to achieving long-term abstinence, and current guidelines specifically recommend starting counseling early after hospitalization for ST segment elevation myocardial infarction.^7^ However, findings from Germany suggest that physicians often fail to adhere to these guidelines,^8^ and even following the implementation of standard operating procedures for the care of smokers hospitalized with cardiovascular disease, the proportion of patients who recalled having received any counseling was well below 50%.^9^ Similarly, suboptimal rates of smoking status documentation and counseling activity have been reported for general practice across Europe.^10^

A lack of training has been suggested as a major barrier preventing physicians from supporting smokers willing to quit.^11^ Ideally, knowledge of the mechanisms underlying nicotine addiction and effective stop smoking medications as well as practical counseling skills should be acquired during undergraduate medical education. Studies from various countries suggest that these issues are not adequately covered in many undergraduate curricula.^12–17^ A nationwide survey of German medical students with approximately 20 000 participants revealed that even in the final year, the proportion of students feeling well prepared to counsel smokers willing to quit was below 10%.^18^

A number of teaching interventions aimed at improving knowledge and skills with regard to smoking cessation among medical students have been developed, but most of these were implemented and evaluated outside Europe.^19,20^ Even across Europe, educational needs differ with regard to the type of support available to patients: While physician counseling can be kept to a minimum in countries operating a network of smoking cessation services, more intense counseling may be needed in countries lacking such a network. As a consequence, teaching interventions should be tailored to the specific needs of the targeted student population.^21^ The aim of this study was to develop and evaluate a short module on tobacco and smoking cessation for undergraduate medical education in Germany. We hypothesized that, compared to students receiving standard didactic teaching, students participating in the novel module would retain more knowledge and practical skills over a period of 6 months.

## Methods

### Design of the Teaching Intervention

Undergraduate medical education at Göttingen University Medical Centre consists of a 2-year preclinical phase and a 3-year clinical phase, followed by 1 year of elective placements before graduation. The new module was designed to be included in a 6-week course on cardiovascular and respiratory disease at the beginning of the second year of the clinical phase. Development of the module was guided by the six-step approach suggested by Kern et al.^22^ Based on the needs identified in the German medical student survey,^18^ a total of 20 specific learning objectives were defined, 13 of which referred to knowledge while 6 referred to practical skills and one to attitudes (see Table 1). Four teaching formats aligned to these learning objectives were identified:

- Podcast: A recording of a 60-min lecture on epidemiology (including smoking prevalence), the mechanisms of nicotine addiction, withdrawal symptoms, the basic principles of counseling, first-line medication, and potential benefits and risks of electronic cigarettes was made available to students on an online repository. Students were invited to watch the MP4 file during 1 week leading up to a plenary session. Students were also encouraged to submit any specific questions to the module organizers via E-mail.
- Lecture: In this 45-min plenary session including a live conference call, an international expert on smoking cessation presented a short lecture on the psychology of tobacco addiction and subsequently discussed the questions submitted by students (see above).
- Seminar: In this 45-min session, groups of 30 students were instructed on counseling according to the 5A approach. In addition, indications, contraindications, and adverse effects of three first-line stop smoking medications (nicotine replacement therapy, bupropion, and varenicline) were discussed.
- Small-group sessions: Small groups consisting of six students each spent 90min role-playing physician–patient interactions. Scenarios covered common conditions (eg, hospitalization for myocardial infarction, smoking in pregnancy, depression, chronic obstructive pulmonary disease). Following each role-play interaction, a psychologist trained in counseling provided individual feedback to students. For preparatory purposes, a sample video of a counseling session (6.5min) was available to students throughout the entire duration of the module.

**Table 1.** Blueprint of the Novel Teaching Module

Learning objective	Teaching	Assessment	Score (%)			*p* Friedman
			T1	T2	T3	
Life expectancy of smokers and nonsmokers	P, L	MC (one item)	50.0±50.4^a^	93.3±25.2^ab^	53.3±50.3^b^	<.001
Mechanisms of nicotine addiction	P, S	TF (six items)	61.1±19.1^cd^	81.1±13.9^c^	77.5±17.8^d^	<.001
Smoking prevalence in Germany	P, L	MC (one item)	18.3±39.0^e^	80.0±40.3^ef^	31.7±46.9^f^	<.001
Annual incidence of quit attempts in Germany	P, L	SAQ (two items)	56.7±39.6	57.5±38.9	62.5±40.8	.484
5A approach to counseling	P, L, S	SAQ (six items)	1.7±5.0^gi^	86.7±23.3^gh^	54.7±35.4^hi^	<.001
NRT: mechanism of action	P, S	MC (one item)	33.3±47.5^jk^	68.3±46.9^j^	65.0±48.1^k^	<.001
NRT: available products	P, S	SAQ (three items)	15.0±18.8^ln^	70.0±26.5^lm^	54.4±26.0^mn^	<.001
NRT: adverse effects	P, S	TF (five items)	45.7±21.2^o^	57.3±23.7^op^	42.7±20.3^p^	.001
Bupropion: adverse effects	P, S	MC (one item)	36.7±48.6	46.7±50.3	35.0±48.1	.303
Varenicline: mechanism of action	P, S	MC (one item)	68.3±46.9	73.3±44.6	58.3±49.7	.080
Varenicline: adverse effects	P, S	MC (one item)	56.7±50.0^q^	73.3±44.6^q^	60.0±49.4	.067
Electronic cigarettes: potential risks and benefits	P, L	TF (six items)	50.8±20.5^rs^	62.8±20.9^r^	57.5±21.4^s^	.003
Withdrawal symptoms	P, S	MC (five items)	91.3±16.6	91.7±15.7	90.3±16.7	.887
Ability to take a full smoking history	RP	OSCE	—			—
Ability to adjust language to individual patient needs	RP					
Ability to deliver structured counseling (5A)	RP					
Ability to tailor counseling to individual patient needs	RP					
Explaining the “not-a-puff” rule for relapse prevention	RP					
Ability to respond to fears expressed by patients	RP					
Considering smoking to be a chronic disease	P, L, S, RP	Survey	—			—

L, lecture; P, podcast; RP, role play; S, seminar. MC, multiple choice question; NRT, nicotine replacement therapy; OSCE, objective structured clinical examination; SAQ, short answer question; TF, true false question. Score (%) refers to student performance in the written exam at T1, T2, and T3, respectively. Different pairs of superscript letters indicate a significant difference (*p* < .05) between two timepoints (Wilcoxon test) for items with significant between-group differences in the Friedman test.

According to current guideline recommendations, cessation counseling should be delivered according to the “5A” approach (ask, advise, assess, assist, and arrange).^23^ More recently, “very brief advice” has been suggested as an alternative to the more time-consuming 5A approach. However, it relies on the availability of stop smoking services where treatment is continued. This approach is more feasible for the general practice setting than the 5A approach, and it has been included in various guidelines.^24,25^ For the purpose of the current study, we focused on the 5A as there is no network of readily available stop smoking services in Germany, hence the need for more intense counseling by physicians.

### Study Design

**Figure 1.**
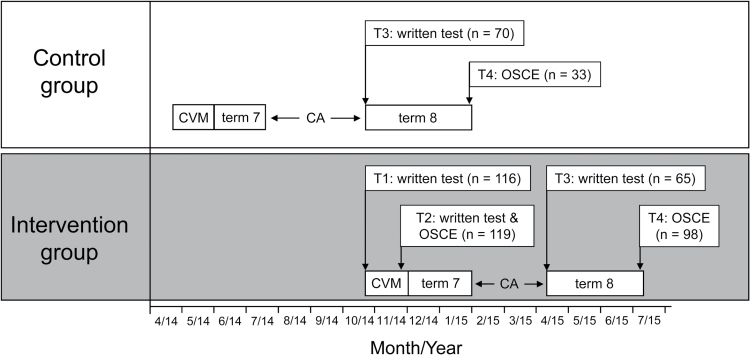
Study outline. CA, clinical attachments; CVM, cardiovascular module; OSCE, objective structured clinical examination. For each data collection point, the number of students providing data is indicated.

This was an observational prospective intervention study with 6-month follow-up. The study design is displayed in Figure 1: Teaching on smoking cessation was delivered during a mandatory 6-week cardiovascular teaching module in the first half of the fourth year (“term 7”) of undergraduate medical education at Göttingen University Medical Centre. Students enrolled in the module in summer term 2014 were selected as a historical control group. They received standard teaching consisting of a 45-min lecture. Students enrolled in the module in winter term 2014/15 were assigned to the intervention group and received more intensive teaching (see below). These students took a written knowledge test at the beginning of term 7 (T1) and another written test as well as an objective structured clinical examination (OSCE) at the end of the cardiovascular module (T2).

Following the cardiovascular module, all students attended teaching modules unrelated to smoking and smoking cessation for a total of 8 weeks, and this was followed by a 2- to 3-month period of clinical attachments during which no formal teaching on tobacco was delivered. Students then entered the second half of the fourth year (“term 8”). At the beginning of this 14-week period (T3), all students (regardless of study group) took a written retention test. At the end of term 8 (T4), retention of practical skills was assessed in an OSCE in all students.

### Assessment of Student Performance

Data collection tools were aligned to the learning objectives listed in Table 1. Knowledge items that were covered in the podcast, lecture, and seminar were addressed in written examinations at T1, T2, and T3. The multiple choice, short-answer, and true/false questions used at all timepoints were identical, yielding a maximum score of 39 points. In addition to the knowledge test, students were surveyed on their current approach to counseling smokers. With regard to attitudes toward smoking, students were asked to rate the statement “I consider smoking to be a chronic disease.” on a six-point scale anchored at 1 (“completely agree”) and 6 (“completely disagree”). Prior to analysis, data were dichotomized by collapsing scale options 1 and 2 into a positive answer.

Practical skills that were trained during small-group sessions in the intervention group were assessed in an OSCE at the end of the cardiovascular module and half a year later. Students in the control group only took the retention OSCE at the end of term 8. In the OSCE, each student was allocated 7min to counsel a standardized patient (lay actors specifically trained for their role) based on a case history that was displayed at the entrance of the consultation room. The case history was identical for all students; it was based on a 54-year-old smoker who had been admitted to hospital with an acute myocardial infarction. Student performance was rated on a checklist aligned to the 5A with a maximum score of 50 raw points available. Raters were trained using this checklist and detailed descriptions of expected skills levels for each item. Questionnaires and checklists are available from the authors upon request.

### Data Collection and Analysis

Paper questionnaires were used for written examinations and surveys at T1, T2, and T3. OSCE raters used paper forms to complete checklists. Questionnaires and checklists were produced with EvaSys (Electric Paper, Lüneburg) and EvaExam (Electric Paper, Lüneburg), respectively. Following the exams, paper forms were scanned and data exported as .cvs files. Data obtained at all timepoints were transferred to SPSS 23.0 (IBM SPSS Statistics) and matched using a self-generated code entered by students.

The primary endpoint of this study was the performance difference between the intervention and control group with regard to knowledge and attitudes (T3) as well as practical skills (T4). All students with valid data at T3 and T4 were included in these analyses. Secondary analyses assessed the increase/decrease of knowledge (T1–T3) and skills (T2 vs. T4) in the intervention group only. Only students with complete data on all relevant timepoints were included in these analyses. Owing to the nonparametric distribution of data, differences between groups were assessed using Mann–Whitney *U* tests, and differences between timepoints within groups were assessed using Friedman and Wilcoxon tests. Differences in dichotomous variables were assessed using χ^2^ tests. Effect sizes were calculated as Cohen’s *d*.^26^ In a sensitivity analysis, the impact of student smoking status, sex, and age on exam performance at T3 and T4 was assessed by including these variables as covariates in a repeated-measures analysis of variance (ANOVA). In the intervention group, separate ANOVAs were performed to assess performance changes between T1 and T3 (written test) and between T2 and T4 (OSCE) with regard to student smoking status, sex, and age. Data are presented as mean ± SD. Significance levels were set to .05. Ethics approval was obtained from the Institutional Review Board at Göttingen Hospital Medical School (application number 15/9/14).

## Results

### Description of the Study Samples

In winter 2014/15, a total of 135 students were enrolled in the cardiovascular module, and 125 agreed to participate in the study (response rate 92.6%). A majority of these were female (60.2%), three out of four were less than 28 years old, and 12.8% were occasional or regular smokers (see Table 2). The number of students providing data differed between data collection points and was lowest at T3 (*n* = 65). Complete data at T1, T2, and T3 were available for 60 students.

**Table 2.** Demographics, Exam, and Survey Results at Follow-up

Parameter	Control group	Intervention group	*p* value
Demographics			
Sex (% female)	73.5	60.2	.064
Age (% <28 years)	75.7	73.7	.759
Smoking status (% smokers)	8.7	12.5	.475
T3 (6 months after teaching)			
T3: written test (% score)	51.7±12.8	61.1±11.7	<.001
T3: “I consider smoking to be a chronic disease” (% agreement)	68.1	83.1	.045
T3: Regular documentation of patient smoking status (%)	95.7	87.7	.018
T3: Quit advice provided to patients (%)	56.1	68.3	.154
T4 (9 months after teaching)			
T4: OSCE—total % score	60.5±10.5	71.5±12.8	<.001
T4: OSCE—Ask (% score)	76.7±12.2	84.1±16.1	.003
T4: OSCE—Advise (% score)	29.0±25.0	40.6±21.6	.005
T4: OSCE—Assess (% score)	96.7±17.4	90.8±29.0	.251
T4: OSCE—Assist (% score)	51.5±13.9	77.6±20.5	<.001
T4: OSCE—Arrange (% score)	69.9±49.7	69.4±46.3	.973

Data are presented as percentages or mean ± SD, as appropriate; *p* values refer to χ^2^ tests and Mann–Whitney *U* tests, as appropriate. OSCE, objective structured clinical examination.

Owing to the smaller number of students enrolled for term 8 modules in summer 2014, only 94 students were eligible for inclusion in the control group, and 70 provided written consent to participate (response rate 74.5%). A majority were female (73.5%) and less than 28 years old (75.7%). Smoking prevalence was similar to the intervention group (8.7%; *p* = .475).

### Knowledge, Attitudes, and Skills in the Two Study Groups at Follow-up

At T3 (ie, 6 months after attending the cardiovascular module), students in the intervention and control group scored 61.1±11.7% and 51.7±13.8% of points in the written knowledge test, respectively (*p* < .001; Cohen’s *d* = .74). Fewer students in the intervention group indicated to regularly document patient smoking status, and there was no evidence of a difference in the proportion of students routinely advising smoking patients to quit (see Table 2). With regard to attitudes, the proportion of students agreeing that smoking was a chronic disease was 83.1% in the intervention and 68.1% in the control group, respectively (*p* = .045). An ANOVA including student smoking status, sex, and age as covariates did not yield significant effects of these variables on student performance in the written test; in the adjusted model, group assignment still had a significant effect (*p* < .001; *η* ^2^ = .118).

At T4 (ie, 9 months after attending the cardiovascular module), students in the intervention and control group scored 71.5±12.8% and 60.5±10.5% of points in the OSCE (*p* < .001; Cohen’s *d* = .90; see Table 2).

Based on a pass/fail cutoff of 60% of available points that is usually applied at German universities, 54.5% of students in the control group would have passed the OSCE while this proportion was 83.7% in the intervention group (*p* = .001). An ANOVA including student smoking status, sex, and age as covariates did not yield significant effects of these variables on student performance in the OSCE; in the adjusted model, group assignment still had a significant effect (*p* < .001; *η* ^2^ = .151).

### Longitudinal Analysis of Student Performance in the Intervention Group

A longitudinal analysis of student performance in all written examinations was performed in a subgroup of students in the intervention groups who provided data at T1, T2, and T3 (*n* = 60). A detailed analysis of student performance with regard to specific learning objectives is provided in Table 1. There was a significant increase followed by a significant decrease in knowledge for some items (including smoking prevalence, impact of smoking on life expectancy, adverse effects of nicotine replacement therapy). For other items, knowledge increased during teaching and remained favorable at follow-up (eg, 5A approach, mechanisms of nicotine addiction, mode of action and types of nicotine replacement therapy, potential benefits and risks of electronic cigarettes). Finally, there was no significant difference in knowledge before and after teaching and at follow-up for some items (eg, incidence of quit attempts, mechanism of action and adverse effects of prescription medication, withdrawal symptoms). With regard to the percent score achieved in the written test at T1 and T3, repeated-measures ANOVAs yielded no significant interaction between student performance and smoking status (*p* for interaction = .082), sex (*p* = .488), and age (*p* = .211).

**Figure 2.**
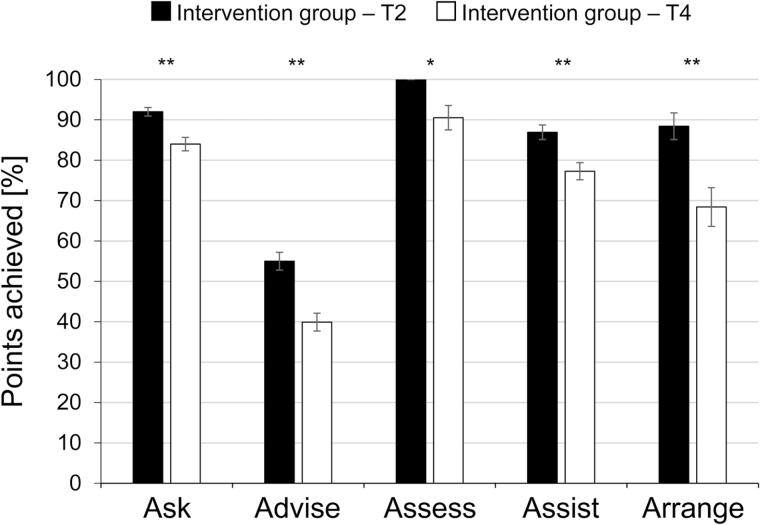
Points achieved by students in the intervention group in the objective structured clinical examination at T2 and T4. Results are presented by the five categories of the 5A approach to cessation counseling. Error bars indicate standard errors of the mean. *p < .05; **p < .001 for comparisons between T2 and T4 (Wilcoxon test).

A longitudinal comparison of OSCE results in the intervention group revealed that between T2 and T4, percent scores dropped significantly for all 5A categories but were still at least 75% for “ask,” “assess,” and “assist” at the 9-month follow-up (see Figure 2). Retention was moderate for the “arrange” category and poor for the “advise” category. Notably, even directly after teaching (T2), student scores in this category were as low as 55%. With regard to the percent score achieved in the OSCE at T2 and T4, repeated-measures ANOVAs yielded no significant interaction between student performance and smoking status (*p* for interaction = .271), sex (*p* = .587), and age (*p* = .670).

## Discussion

This intervention study found a significant and meaningful association of the teaching intervention with student knowledge, attitudes, and skills regarding smoking cessation counseling, compared with a historical control group. Compared to the control group, retention of knowledge and skills was higher in the intervention group by a large effect size. However, there was no evidence of improved self-reported practice in the intervention group compared with the control. The intervention was feasible, short, and aligned to the needs of the German healthcare system.

No significant improvement was observed for some items in the knowledge test, and given that initial knowledge was favorable even before the teaching intervention, efforts could be shifted toward items with moderate medium-term retention despite their relevance for clinical practice (eg, adverse effects of medication, potential risks and benefits of electronic cigarettes). This might be achieved by pitching the cases used in practical skills training sessions to these needs. For example, scenarios could include smokers who are currently trying to quit and are experiencing medication side effects. Also, feedback instructions for session facilitators could put more emphasis on those items for which a suboptimal learning outcome was observed.

Retention of practical skills as measured in the OSCE was generally favorable. It has been shown that patient satisfaction with primary care is higher the more of the 5A are covered during a consultation.^27^ A more recent case–control study found that delivery of “assist” and “arrange” significantly increased the odds of quitting within 6 months.^28^ Brief advice is associated with an increase of quit attempts even in patients not willing to quit.^29^ Thus, our finding of low initial coverage and low retention of skills related to the second A (“advise”) points to a need to further improve the teaching intervention with a specific focus on this aspect. This is particularly relevant for patients with cardiovascular disease as the presenting complaint may serve as an ideal peg on which to hang counseling activities. Given the association between smoking and respiratory and malignant disease,^30,31^ the implications of our study extend to this patient group too.

### Comparison With Earlier Studies

A number of teaching interventions on tobacco-induced diseases and approaches to smoking cessation have been implemented and evaluated,^19,20^ and the heterogeneity of studies (including outcome measures) precludes direct comparisons. In addition, most of these trials were performed in the United States. Given the difference between healthcare systems, these approaches cannot be readily transferred to European countries. The curriculum described here was tailored to the German healthcare system in which cessation medication is not covered by health insurers and there is no national network of stop smoking services.

A recent systematic review of behavior change counseling curricula^32^ that also featured 67 studies on smoking cessation concluded that, in order to be effective, teaching interventions should be based on existing frameworks (eg, the 5A) and learners should be offered feedback during practical skills training. Successful curricula also employed diverse activating teaching methods. The novel module described here used innovative methods including the flipped classroom approach where student learning is stimulated with preparatory material, thereby freeing up lecture time for interaction and discussion rather than knowledge transfer.^33^

### Strengths and Limitations

This study was based on current recommendations for the design of teaching interventions^32^; and according to Kirkpatrick’s hierarchy of outcome levels,^34^ the exams and questionnaires used in this study covered the modification of knowledge and skills (level 2b) and behavioral change itself (level 3). In addition, we used a prospective design and included a (historical) control cohort. Assessment methods were aligned to learning objectives, and participation in the intervention was mandatory for all students enrolled in the cardiovascular module. Interpretation of our findings is limited by the monocentric design of our study, the fact that the teaching module was tailored to the needs of one national healthcare system and the considerable drop-out observed at T3.

The size of the longitudinal samples used to assess changes in knowledge (*n* = 60 students) and skills (*n* = 95 students) within the intervention group was acceptable, and we did not find significant differences regarding smoking prevalence, sex, and age between students providing data and students not providing data for any of the four data collection points (data not shown). Since baseline data in the control group were not collected at T1 but at T3, we cannot comment on potential group differences at baseline (ie, start of term 7). However, the data presented in Table 2 do not indicate any significant between-group differences at T3.

Total learning time was unequal in the two groups (control: 45-min lecture; intervention: podcast, lecture, seminar, small-group teaching, and supplemental video material). Increased exposure to the material to be learnt may by itself increase learning outcome. In addition, current research on the impact of repeated testing on long-term retention^35^ suggests that the additional testing that occurred in the intervention group may have further increased learning outcome in the intervention group. We are unable to differentiate whether the improvement in outcomes occurred due to these specific interventions themselves or additional exposure time in the intervention group. In order to achieve a similar effect in other student populations, the teaching module (including repeated formative testing) should be implemented in its entirety. Finally, we did not measure patient effects.

### Implications for Future Research

Future studies should assess whether students participating in the novel teaching module deliver more and more effective counseling to their patients. Given the necessity to keep costs for educational activities at a reasonable level, a cost-effectiveness analysis comparing the investment in time and resources for teaching with the potential benefit for patients (measured as life-years saved) would be helpful.

## Conclusions

The novel teaching module had significant and meaningful effects on medium retention of student knowledge, attitudes, and skills. Future trials should address the impact of the teaching intervention on patient satisfaction and quitting success.

## Funding

There was no specific funding for this study. JB’s post is funded by a fellowship from the Society for the Study of Addiction and Cancer Research UK also provided support (C1417/A14135).

## Declaration of Interests

*TR has received honoraria from Pfizer, Novartis, Glaxo Smith Kline, Astra Zeneca, and Roche as a speaker in activities related to continuing medical education. He has also received financial support for investigator-initiated trials from Pfizer and Johnson & Johnson. JB has received unrestricted research grants from Pfizer. AB has received sponsorship to attend scientific meetings and to speak in activities related to medical education from Astra Zeneca, Boehringer Ingelheim, Glaxo Smith Kline, Novartis, and Pfizer. AME has received travel funding, honorariums, and consultancy payments from manufacturers of smoking cessation products (Pfizer, Novartis, and Glaxo Smith Kline) and hospitality from North51 who provide online and database services. He also receives payment for providing training to smoking cessation specialists, receives royalties from books on smoking cessation, and has a share in a patent of a nicotine delivery device. All other authors have no conflict of interest to declare.*
